# *In Utero* Programming of Later Adiposity: The Role of Fetal Growth Restriction

**DOI:** 10.1155/2012/134758

**Published:** 2012-11-01

**Authors:** Ousseynou Sarr, Kaiping Yang, Timothy R. H. Regnault

**Affiliations:** ^1^Department of Obstetrics and Gynaecology, Children's Health Research Institute and Lawson Research Institute, University of Western Ontario, 1151 Richmond Street, London, ON, Canada N6A 5C1; ^2^Dental Science Building, Room 2027, University of Western Ontario, 1151 Richmond Street, London, ON, Canada N6A 5C1

## Abstract

Intrauterine growth restriction (IUGR) is strongly associated with obesity in adult life. The mechanisms contributing to the onset of IUGR-associated adult obesity have been studied in animal models and humans, where changes in fetal adipose tissue development, hormone levels and epigenome have been identified as principal areas of alteration leading to later life obesity. Following an adverse *in utero* development, IUGR fetuses display increased lipogenic and adipogenic capacity in adipocytes, hypoleptinemia, altered glucocorticoid signalling, and chromatin remodelling, which subsequently all contribute to an increased later life obesity risk. Data suggest that many of these changes result from an enhanced activity of the adipose master transcription factor regulator, peroxisome proliferator-activated receptor-*γ* (PPAR*γ*) and its coregulators, increased lipogenic fatty acid synthase (FAS) expression and activity, and upregulation of glycolysis in fetal adipose tissue. Increased expression of fetal hypothalamic neuropeptide Y (NPY), altered hypothalamic leptin receptor expression and partitioning, reduced adipose noradrenergic sympathetic innervations, enhanced adipose glucocorticoid action, and modifications in methylation status in the promoter of hepatic and adipose adipogenic and lipogenic genes in the fetus also contribute to obesity following IUGR. Therefore, interventions that inhibit these fetal developmental changes will be beneficial for modulation of adult body fat accumulation.

## 1. Introduction

Obesity refers to excessive adipose tissue accumulation and is defined by the World Health Organization (WHO) as a body mass index (BMI: weight (kg)/length (m^2^)) greater than or equal to 30 [1]. Obesity has been declared a major health problem and its incidence has more than doubled worldwide since 1980 with over 200 million men and nearly 300 million women being classified as obese in 2008 according to the WHO. Obesity is associated with numerous adverse health consequences, including type 2 diabetes, insulin resistance, hypertension, cardiovascular disease, and certain cancers [2, 3]. The direct costs associated with obesity were estimated to account for between 0.7% and 2.8% of a country's total healthcare expenditures with medical costs of obese individuals being approximately 30% greater than their normal weight peers [4]. Thus, social and economic costs related to obesity in developed countries are now well recognized. 

It has been reported that the current intervention strategies to prevent and manage obesity and its associated diseases are limited to postnatal life with focus on exercise, salt intake, dietary interventions, and smoking cessation [5]. These interventions have limited success and it is not surprising that the battle against obesity and its associated diseases particularly in wealthy industrialized countries is currently being lost. Gluckman and Hanson [5] suggest that it is important to refocus on maternal health and nutrition issues during pregnancy, which are now considered to play a major role in the onset of obesity. 

In this review we summarize epidemiological and animal studies linking adverse *in utero* environments, particularly IUGR, to postnatal adipose tissue accumulation. We also highlight potential mechanisms underlying links between IUGR and the long-term adipose tissue expansion and emphasize some ideas for further research in IUGR models.

## 2. The Fetal Programming Concept

The term programming in the broad sense was suggested by Lucas [6], to name the process by which a stimulus or insult during critical periods of life results in long-term consequences such as induction, deletion, or impairment of a somatic structure or alteration of a physiologic function. Earlier animal experiments reported the early environment to be a major determinant of growth and form [7]. Human cohort studies also reported an inverse association between birth weight and systolic blood pressure in 36-year-old men [8]. It was in the early 80s that the “fetal programming” and “early life origins of adult diseases” concepts as proposed by David Barker and colleagues really began to cement the importance of the *in utero* environment. Barker and colleagues proposed that environmental factors, particularly nutrition, act in early life to program the onset of cardiovascular disease in early adult life and premature death as the consequence [9]. This association has been postulated to be an adaptive response to a suboptimal fetal environment protecting the growth of key organs such as brain to the detriment of others such as liver and resulting in an altered postnatal metabolism. These adaptations, termed the “thrifty phenotype” [10], serve the purpose of enhancing prenatal survival under conditions of intermittent or poor nutrition [11]. However when nutrition is more abundant in the postnatal environment than in the prenatal environment, the changes adopted by the fetus before birth may lead to a nutritional mismatch between energy intake, storage, and expenditure, resulting in a subsequent increase in disease risk [11]. Fetal programming is a concept that thus identifies *in utero* environmental conditions as key determinants for the increased risk of diseases later in life. Epidemiological observations as well as clinical and animal studies worldwide support the concept of fetal programming as the origin of a number of diseases including obesity, insulin resistance, and noninsulin-dependent diabetes [12–15]. Specifically, the “early life origins of obesity” concept has led to the hypothesis that exposure to excessive or deficient nutrition before birth alters the development of the fat cell, the adipocyte, *in utero* and results in a permanent increase in the capacity to form new cells in adipose depots or to store lipid in existing adipocyte in postnatal life [16].

## 3. Adipose Tissue

### 3.1. The Different Types of Adipose Tissue

Two types of adipose tissue, white adipose tissue (WAT) and brown adipose tissue (BAT), coexist in most mammalian species. WAT has an essential role in energy storage by providing long-term fuel reserve in the form of triacylglycerols, which can be mobilized during food deprivation with the release of fatty acids for oxidation in others organs [18]. BAT, on the other hand, is specialized in the dissipation of energy through the production of heat [19]. 

The WAT is made up of unilocular adipocytes, which contain a single large lipid vacuole that pushes the cell nucleus against the plasma membrane [20]. The biogenesis of white adipocytes comprises the generation of committed adipocyte precursors (or preadipocytes) and the terminal differentiation of these preadipocytes into mature functional adipocytes [21]. This is accompanied by the expression of adipogenic and lipogenic transcription factors including peroxisome proliferator-activated receptor-*γ* (PPAR*γ*), PPAR*δ*, CCAAT/enhancer binding proteins (C/EBP*α*, *β*, *δ*), and the sterol regulatory element-binding protein 1 (SREBP1) and the expression of specific lipid-metabolizing enzymes such as FAS [22–26]. These transcription factors appear to be part of a cascade in which PPAR*γ* is the master regulator with its activity modulated by selecting corepressors and coactivators including SRC1 (steroid receptor coactivator 1), SIRT1 (an NAD^+^-dependent histone deacetylase and chromatin-silencing factor), NCoR (nuclear receptor corepressor), and SMRT (silencing mediator for retinoid and thyroid hormone receptor) [27, 28]. Following this gene regulation cascade, the adipogenesis process ends with the establishment of the endocrine function characterised by the production of the adipocyte-specific hormone, leptin [29]. Leptin circulates at levels proportional to body fat and acts on the central nervous system to regulate energy intake and expenditure, through binding with neuropeptide Y (NPY) neurons producing a feeling of satiety. 

In mammals, WAT is distributed unevenly through the body and is represented by two main fat depots, which are defined by their location: subcutaneous and visceral [30]. In humans, subcutaneous depots consist of adipose tissue under the skin in primarily the buttocks, thighs, and abdomen. Visceral adipose tissue depots include the mesenteric, omental, perirenal, retroperitoneal, and pericardial fat stores [31]. In sheep, a large animal model of adult onset obesity, WAT is present in the omental, subcutaneous and hindlimb regions [32–34]. WAT depots in rodents (rats and mice), exist in two main subcutaneous fat depots, one anterior and one posterior, lying in discrete anatomical sites [35]. The anterior depot is complex, occupying the dorsal body region between and under the scapulae, the axillary and proximal regions of forelimbs, and the cervical area. The posterior depot is located at the base of hind legs and at dorsolumbar, inguinal, and buttock regions. The visceral adipose depots similarly to humans, are located in thoracic and abdominal cavities: mediastinic, mesenteric, retroperitoneal, perirenal, and perigonadal depots. 

The second type of adipose tissue, the BAT, is specialized in the dissipation of energy through the production of heat [19]. It is characterised by having a dark color compared to WAT, which arises from its vascularization and numerous mitochondria [36, 37] and appears to have a denser nerve supply than WAT [38]. In BAT, multilocular adipose cells usually contain many small vacuoles of lipid and large mitochondria with closely packed parallel cristae [39, 40], where the uncoupling protein 1 (UCP1) is highly expressed and is regarded as a BAT-specific marker [41]. In conjunction with UCP1, a number of other genes including type 2 iodothyronine deiodinase, the transmembrane glycoprotein Elovl3, the fatty-acid-activated transcription factor peroxisome proliferator-activated receptor-*α* (PPAR*α*), the nuclear coactivator peroxisome proliferator-activated receptor-*γ* coactivator 1*α* (PGC-1*α*), and developmental homeobox genes HoxA1 and HoxC4 are preferentially expressed in BAT [37, 42]. By way of comparison, expression of leptin, the nuclear corepressor RIP140, matrix protein fibrillin-1, and developmental human genes HoxA4 and HoxC8 in BAT are low compared to their greater expression observed in WAT [37, 42].

It has long been assumed that white and brown adipocytes share a common developmental origin and also undergo a very similar program of morphological differentiation controlled by PPAR*γ* and members of the C/EBP family of transcription factors [43]. However, recent studies indicate that brown adipocytes arise from tripotent engrailed-1-expressing cells in the central dermomyotome through a dynamic involvement of the PRD1-BF-1-RIZ1 homologous domain-containing protein-16 (PRDM16) [43, 44]. In addition, PRDM16 coactivates the transcriptional activity of PGC-1*α*, PGC-1*β*, PPAR*α*, and PPAR*γ* through direct interaction and thus drives preadipocytes development into brown adipocytes [43]. This differential origin is probably determinative for the evolutionary role of BAT and WAT in mammals.

In the human fetus and newborn, BAT is located mainly in the cervical, axillary, perirenal, and periadrenal depots [45, 46] and plays an important role in nonshivering heat production during neonatal life and thus provides protection against lethal cold exposure (hypothermia). In adults, the depots of BAT are found in a region extending from the neck to the thorax, especially in interscapular, supraclavicular, cervical, axillary, and paravertebral regions [47, 48] and these depots are now understood to be associated with body weight regulation [49]. In comparison, BAT in rodents is located mainly in the upper back region (interscapular BAT) [50] and first appears during the last days of gestation, matures during the neonatal period, and remains at a relatively stable level for the life span of animals [51]. BAT is also visible in the subcutaneous anterior depot and mediastinic and perirenal sites in adult rodents maintained in normal conditions [35]. In other species, the situation is quite different. For example, lambs are born with almost 100% BAT [52, 53], with majority of this adipose tissue located around the kidneys [33, 34]. Postnatally in young life, BAT localization becomes the sternal, clavicular, pericardial, and epicardial depots in addition to the perirenal depot [34]. 

### 3.2. Ontogeny of Adipose Tissues

**Figure 1:**
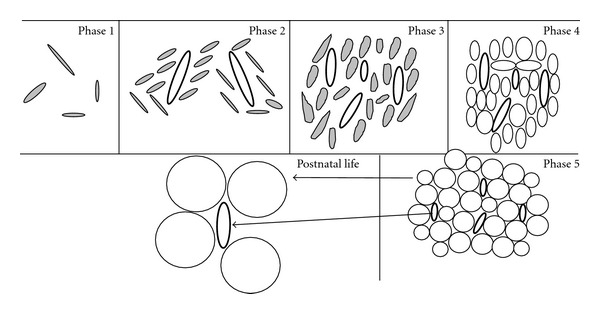
Developmental stages of adipose tissue (adapted from Brooks and Perosio, [17]). Phase 1: emergence of loose connective tissue composed of an amorphous ground substance and stellate cells (filed). Phase 2: aggregates of mesenchymal cells (filed) are condensed around proliferating primitive blood vessels (bold ovals). Phase 3: mesenchymal cells differentiating into stellate preadipocytes within a glomerulus. Phase 4: appearance of adipocytes with multiple small lipid droplets closely packed around the capillaries. Phase 5: fat lobule with many unilocular cells (clear circles) is evident. This developmental process (phase 1 to 5) occurs between the 14- and 23-week gestation period. From 23 to 29 weeks, the number of fat lobules is relatively constant. From the 23rd to 29th week and throughout postnatal life, the growth of adipose tissue is determined mainly by an increase in size of the fat lobules arising from adipocyte hypertrophy and enlargement of adipose capillaries.

Adipocytes in WAT are generally described to be derived from mesenchymal stem cells (MSCs). These themselves are thought to arise from mesoderm, although an alternative source of MSCs, as well as adipocytes, from the neural crest has recently been demonstrated [21]. Adult adipose tissue develops as a continuous process; however, prenatal adipose tissue formation can be divided into five morphogenic phases strongly associated with the formation of blood vessels (Figure 1). These five stages include (1) the emergence of loose connective tissue, (2) proliferation of primitive vessels associated with mesenchymal condensation, (3) mesenchymal cells differentiating into stellate preadipocytes within a vascular structure or glomerulus, (4) appearance of fine fat vacuoles in cell cytoplasm of mesenchymal lobules, and (5) fat lobules well separated from each other by dense septae of perilobular mesenchymal tissue [54]. Fat lobules are the earliest structures to be identified before the appearance of typical vacuolated fat cells [55]. In humans, white fat lobules appear first in the face, neck, breast, and abdominal wall at 14 weeks gestation [55]. By 15 weeks, they are also evident over the back and shoulders and further development of white fat lobules in the upper and lower extremities and anterior chest begins around this time. After the 23rd week, the total number of fat lobules remains approximately constant, while from the 23rd to 29th week, the growth of adipose tissue is determined mainly by an increase in size of the fat lobules.

In comparison, three distinct stages of prenatal WAT differentiation are postulated in rats [56]. In stage 1, a sparse network of large capillaries develops. In stage 2, most of cells are spindle-shaped cells and surrounding connective tissue contains very few blood vessels followed by capillary bed formation. Stage 3 is characterized by a mature capillary bed and rounded adipocytes. The earliest embryonic subcutaneous adipose cells are detected at days 15-16 of gestation (length of gestation ~21–23 days) [57]. Perirenal adipose tissue in rat appears mainly around birth, that is, 12 hours before and after birth [58]. Only two to five days separate the formation of first perirenal adipose cells and the appearance of mesenteric fat cells that develop the last. As a consequence, minimal amounts of adipose tissue (1%) are deposited prior to birth and maturation of this tissue primarily occurs postnatally [59].

In rats the brown adipocyte precursors are parenchymal spindle cells closely related to a network of capillaries [60]. As the cells and vessels proliferate, they are organized into lobules by connective tissue septa. When the cells start accumulating lipid, they initially are unilocular, but with further lipid accumulation, multiple cytoplasmic lipid vacuoles appear. BAT formation takes place in the scapula of rats between day 15 to 17 of gestation [60, 61] and is present throughout life [50]. Human studies are not as specific as in rats; however studies suggest that fetal BAT is observed in the cervical, thoracic, and abdominal viscera and at the shoulder girdle and neck at approximately 23 weeks of pregnancy [62].

In the postnatal environment, expansion of adipose tissue occurs mainly after birth through increases in adipocyte size and enlargement of adipose capillaries (Figure 1) under the actions of enzymes such as lipoprotein lipase, a regulator of adipocyte lipid filling [63, 64]. Adipocyte hyperplasia following birth appears limited; however studies do report its activation for the renewal of adipocytes [65] suggesting that WAT and BAT in humans, as well as in rodents, still contain precursor cells capable of differentiating into adipocytes at adulthood [66–68]. 

## 4. Long-Term Consequences of IUGR on Adipose Tissue Development

IUGR or fetal growth restriction (FGR) which refers to a fetus that fails to meet its genetic growth potential, is characterized by a weight at or below the 10th percentile for gestational age and affects approximately 7–15% of pregnancies worldwide [69]. The association between IUGR and the postnatal development of obesity has been reported in human epidemiological studies and in animal models [70, 71] and their interaction is postulated to be a major contributor to the current global obesity epidemic [5, 70].

### 4.1. Effects of IUGR and Low Birth Weight on Long-Term Adipose Tissue Expansion in Animal Models

A number of animal models have been developed to examine the effects of *in utero* insults such as maternal undernutrition and placental insufficiency on the long-term adipose tissue expansion and function. In the frequently used rodent maternal low-protein model (50% protein restriction during gestation), IUGR and subsequent obesity have been reported [14, 72–75]. While protein restriction in pregnancy itself is sufficient to lead to obesity, this effect is enhanced by overfeeding during the suckling, proving the concept of the nutritional mismatch [74–76]. Further, maternal undernutrition as a nutritional manipulation is characterized by a global dietary restriction during pregnancy and also results in low weight at birth and later obesity in rats [77]. In pigs, low protein diet (6% protein versus 12%) throughout pregnancy results in decreased body weight of piglets at birth and increased WAT percentage at 188 days of age [78]. Moreover, IUGR occurs spontaneously in pigs and these low-birth-weight piglets also display significant higher body fat at 12 months compared to normal-birth-weight piglets [79], highlighting common mechanisms at play between a reduced protein supply *in utero* and a reduced placental exchange capacity as occurs in spontaneous IUGR [80]. In addition, placental insufficiency results in reduced birth weight, increased early postnatal growth, and increased visceral adiposity in adolescent sheep and in young and adult rat offspring [81, 82].

The idea that BAT deposition may change in response to suboptimal *in utero* environment as IUGR and that this adaptation is perpetuated through the life cycle, thereby suppressing energy expenditure and ultimately promoting later obesity, is currently emerging. In the sheep model, placental restriction alters feeding activity, which increases with decreasing size at birth and is predictive of increased postnatal growth and adiposity including the perirenal adipose tissue [83], a depot that displays characteristic of BAT in young sheep [34]. Prenatal nutrition regulates BAT development as studied in fetuses from arginine-treated underfed ewes compared with fetuses from saline-treated underfed ewes [84]. Existing data indicate that nutrient availability during the intrauterine life, independently of fetal growth, determines BAT development and the control of energy utilization during postnatal life period. Indeed, it has been demonstrated that feeding pregnant mice with the low-protein diet throughout gestation results in an unchanged BAT mass and a significantly increased expression of UCP1 in interscapular brown adipose tissue in adult female offspring when compared to normal offspring [85]. It should be noted that in this study, the protein restricted offspring did not display a reduced fetal growth or low birth weight. In contrast, in a female rat offspring born with normal weight, the intrauterine malnutrition resulted in lower BAT deposition accompanied with an increased WAT adiposity at 53 days of age [86]. The programming of BAT is therefore an exciting area that warrants further studies into the effects of IUGR or low birth weight upon postnatal BAT growth and metabolism. 

### 4.2. Human Low Birth Weight and Later Adipose Tissue Accumulation

The first studies addressing low birth weight as a result of fetal growth restriction leading to the subsequent expansion of adipose tissue in adults utilised data obtained from the studies of the offspring born following the Dutch famine of 1944-1945 [87]. Exposure to the famine during the first half of pregnancy resulted in low birth weight and this was significantly associated with higher obesity rates and more truncal and abdominal fat distribution in men at 19 years of age. A subsequent study of this cohort reported a higher BMI and waist circumference in 50-year-old women exposed to the famine in early gestation (first trimester) compared to nonexposed women [12]. The association between low birth weight and later adiposity is also highlighted by studies in a biethnic population (Mexican-American and non-Hispanic white) in the United States. In these studies, normotensive and nondiabetic adult individuals whose birth weight was in the lowest tertile have a significantly greater truncal fat deposition pattern (+14%, measured through the subcapsular-to-triceps skinfold ratio) than individuals whose birth weight was in the highest tertile independently of sex, ethnicity, and current socioeconomic status [88]. 

## 5. Intrauterine Mechanisms behind *In Utero* Programming of Later Adiposity

**Figure 2:**
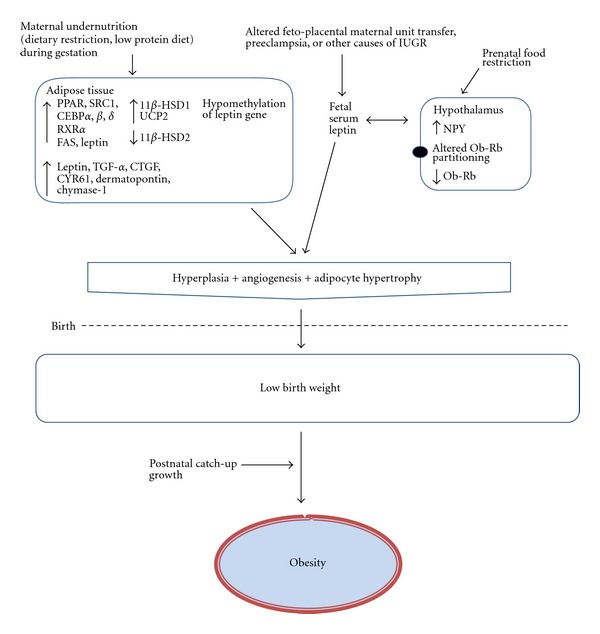
Schematic overview depicting key postulated molecular changes in adipose tissue and in hormonal status in the fetus and that may be involved in the development of later obesity following intrauterine growth restriction. For full explanation and definitions, see (Section 5). TGF-*α*1 (transforming growth factor alpha-1), CTGF (connective tissue growth factor), CYR61 (cysteine-rich, angiogenic inducer, 61), dermatopontin, and chymase-1.

Animal and human studies have focused on several intrauterine mechanisms that may program the fetal adipose tissue for later obesity. Specifically, changes in fetal adipose tissue morphology and metabolism, altered pathways regulating appetite, and modification of hormone levels and epigenome in the fetus have been highlighted as critical regulators in the development of obesity following IUGR (Figure 2). 

### 5.1. The Role of Fetal Adipose Development in the Later Expansion of Adipose Tissue

Emerging evidence from animal studies indicates that an increased prenatal adipocyte differentiation and lipogenesis likely promotes the development of later obesity in IUGR offspring [28, 89]. Such effects imply an early induction of adipose PPAR*γ* activity concomitantly with an upregulated expression of its coactivator SRC1 and its downstream regulatory transcriptional factors (CEBP*α*, *β*, *δ*, and the retinoid X receptor *α*) and a downregulation of hormone-sensitive lipase (HSL), an enzyme favouring adipocyte lipid release [28, 90]. In pigs, metabolic pathways have been identified that underlie early subcutaneous adipose tissue adaptation to prenatal maternal low-protein diet and cause later fattening phenotype [91]. These data indicate that maternal diet restriction during gestation leads to IUGR, affects fetal adipose tissue development and programs its later phenotype. In these experiments, 1-day-old piglets prenatally exposed to low-protein diet displayed an upregulation of proteins involved in the conversion of glucose into fatty acids (e.g., transaldolase 1, aldolase C, enolase 1, and pyruvate dehydrogenase) as well as an increased FAS activity in subcutaneous adipose tissue [91]. In addition, a decreased insulin-like growth factor 1 mRNA expression has been demonstrated in perirenal visceral adipose tissue from placental restriction in sheep fetuses at day 145 of gestation [92], which may alter adipocyte proliferation and differentiation [93], increasing their susceptibility for increased visceral adipose tissue in later life. Moreover, an increased abundance in the expression of genes, involved in adipogenesis (e.g., CEBP-*β*, -*δ*, and FAS) and angiogenesis (e.g., leptin TGF*α*-1, CTGF, CYR61, dermatopontin, and chymase-1) in adipose tissue (Figure 1) as molecular mechanisms that underlie the early programming of later increased visceral adiposity in rats by maternal protein restriction, has been reported [94]. These data emphasize the involvement of prenatal adipose tissue development in later life adult obesity. It is however necessary to note that although an altered metabolism and morphology of adipose tissue during fetal life participates as a mechanism in later obesity related to IUGR, rapid postnatal catch-up growth is also a contributor in such increased adiposity [74, 75]. Indeed, prenatal growth trajectory in conjunction with rapid growth in early infancy (catch-up growth) must be considered to ultimately determine the origins of later diseases such as obesity [95]. 

### 5.2. Leptin, IUGR and Later Adipose Tissue Development

Leptin, a 16 kDa protein hormone, stimulates a negative energy balance by increasing energy expenditure and reducing food intake [96]. Leptin mainly acts by binding to specific central and peripheral receptors in the hypothalamus, adipose tissue, liver, and pancreatic *β*-cells [97]. Studies have highlighted the importance of prenatal leptin in developmental programming of adipose tissue and several human studies have reported that fetal serum leptin levels are lower in IUGR babies [98–101]. Thus, leptin may play a role in the control of substrate utilization and in the maintenance of fat mass before birth, producing permanent changes resulting in adiposity in adulthood [102, 103]. Supporting this idea, it has been demonstrated that neonatal leptin treatment of IUGR piglets and pups reverses high level of fetal cell proliferation in adipose tissue induced by IUGR as well as the associated later increased adiposity [104, 105]. It is possible that in IUGR, the underlying mechanisms of *in utero* leptin action in the developing susceptibility to adult obesity are alterations of the expression of appetite stimulating neuropeptides, such as NPY in the fetal brain [103], alterations in adipose sympathetic innervations [106], as well as an altered hypothalamic leptin receptor (ObRb, obese receptor b) expression and partitioning among the different hypothalamic nuclei [107]. Indeed, ObRb, which is preferentially localized in the arcuate nucleus (ARC) in animals with normal body weight, was found to be almost equally distributed between ARC and paraventricular nuclei (PVN) in IUGR newborn piglets. In addition, a lower expression of ObRb in the ARC of IUGR versus control piglets was observed suggesting a lower sensitivity to leptin action in IUGR leading to altered food intake behaviour and subsequent obesity [107]. In line with that data, leptin administration in both pregnancy and lactation has been shown to provide long-term protection from early maternal low-protein-associated obesity in rats [108].

### 5.3. *In Utero *Exposure to Glucocorticoids and Postnatal Adipose Tissue

The hypothalamo-pituitary-adrenal (HPA) axis has been proposed to participate in the pathophysiology of later life obesity following being born IUGR [109]. The mechanisms are ill defined, but evidence from animal studies suggests that adverse events in early life may influence the neuroendocrine development of the fetus resulting in long-term alterations in the setpoints of several major hormonal axes, including an increase in adrenal glucocorticoid secretion. Indeed, the adipose tissue from early nutrient-restricted sheep fetuses displays alterations in glucocorticoid signalling (increased glucorticoid receptor and 11-*β*-hydroxysteroid dehydrogenase 1 (11*β*-HSD1) expression, but decreased 11*β*-HSD2 abundance) at day 140 of gestation and at 6 months postnatally [110]. As 11*β*-HSD2 converts cortisol to its inactive metabolite cortisone [111] and is thought to protect certain tissues from excess cortisol exposure [112], these results suggest that glucocorticoid action may be enhanced in offspring exposed to nutrient restriction *in utero*, thereby increasing their susceptibility to later obesity. Thus, it has been suggested that this *in utero* increased adipose glucocorticoid sensitivity observed near term in maternal nutrient-restricted sheep fetuses, may subsequently lead to the pathophysiological development of visceral obesity in later life by triggering the acquisition of white adipose tissue characteristics postnatally [110].

### 5.4. Fetal Epigenome and Postnatal Adipose Development

Epigenetic modifications alter gene expression without changes in DNA sequences [113]. Epigenetic systems include DNA methylations, histone modifications, and microRNAs. Low levels of DNA methylation, particularly at gene promoter regions, have been proposed to generate active genes [114]. Elevated DNA methylation at promoter regions may however deactivate genes. As the epigenome is established early in development, during a window in which environmental insults such as *in utero* stress are able to influence developmental trajectories, altered epigenetic regulations are therefore mechanisms which could underlie programmed adiposity in the offspring. The study of altered chromatin structure in IUGR, as it relates to later life obesity, is a new and rapidly evolving field. In maternal low-protein animal models of later life obesity, alterations of the methylation status in the promoter of metabolic genes, such as hepatic PPAR*α*, glucocorticoid receptor (GR), and liver X receptor (LXR) and hypomethylation of leptin promoter in adipose tissue have been reported during fetal and postnatal life [115], highlighting the importance of *in utero* environment as a predeterminant of later life chromatin function. In human studies, investigations of blood samples from the Dutch Hunger Winter cohort at the age of 60 years, report an increased DNA methylation induced by periconceptional exposure to the famine in genes known to be involved in adipose tissue metabolism, specifically leptin and the fat mass and obesity associated gene (FTO) [116] suggesting a possible suppression of its activity. Indeed, modifications in FTO gene expression are reported to modulate tissue lipid metabolism [117], and content [118, 119] as well as lipotoxicity [120] and may be mediated by changes in energy balance at any stage of fetal development. 

## 6. Conclusion and Perspectives

This paper provides a frame work for how adipogenesis and lipogenesis processes may be altered in IUGR and low birth weight, setting the stage for obesity later in life. It presents evidence from both animal and human studies indicating that an increased lipogenic and adipogenic capacity of adipocytes, hypoleptinemia, altered glucocorticoid signalling, and epigenetic modifications during fetal life likely play major roles in the *in utero* origins of later life obesity. Given that discrete molecular changes in fetal adipose tissue have been shown to adversely affect adipose tissue development of IUGR individuals later in life, there is a real need to undertake longitudinal studies (before birth, during early postnatal life, and adulthood) on adipose tissue development and establish definitively which genes and pathways in this tissue have a causal role in the *in utero* origins of obesity.
